# Hybrid hollow silica particles: synthesis and comparison of properties with pristine particles†

**DOI:** 10.1039/d0ra02888f

**Published:** 2020-06-10

**Authors:** Jaswinder Sharma, David A. Cullen, Georgios Polizos, Kashif Nawaz, Hsin Wang, Nitin Muralidharan, David Barton Smith

**Affiliations:** Roll-to-Roll Manufacturing Group, Energy and Transportation Science Division, Oak Ridge National Laboratory Oak Ridge TN 37831 USA sharmajk@ornl.gov +1-(865)241-2333; Center for Nanophase Materials Sciences, Oak Ridge National Laboratory Oak Ridge TN 37831 USA; Building Technologies Research and Integration Center, Oak Ridge National Laboratory Oak Ridge TN 37831 USA; Materials Science and Technology Division, Oak Ridge National Laboratory Oak Ridge TN 37831 USA

## Abstract

In the past decade, interest in hollow silica particles has grown tremendously because of their applications in diverse fields such as thermal insulation, drug delivery, battery cathodes, catalysis, and functional coatings. Herein, we demonstrate a strategy to synthesize hybrid hollow silica particles having shells made of either polymer-silica or carbon–silica. Hybrid shells were characterized using electron microscopy. The effect of hybrid shell type on particle properties such as thermal and moisture absorption was also investigated.

## Abstract 2

Hybrid hollow silica particles, which show different properties compared to their pristine counterparts, have been synthesized.

## Graphical abstract

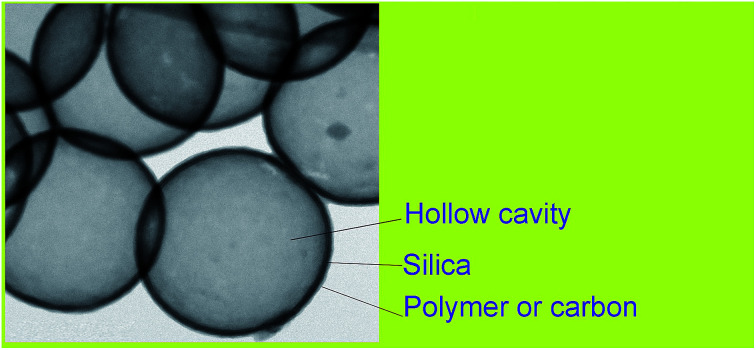

In the past decade, hollow particles have attracted a great deal of interest because of their unique properties (*e.g.*, high surface area, low density, and encapsulated cavity) compared with their dense counterparts. Hollow particles of several materials, including polymers, silica, titania, carbon, and zinc oxide have been reported.^1–9^ Among these, hollow silica particles have attracted great attention from scientists because of their low material cost; well understood chemistry; and potential applications in widespread areas such as thermal insulation, drug delivery, energy storage, phase change encapsulation, catalysis, and superhydrophobic coatings.^10–18^ Hollow silica particles can be synthesized using various approaches, such as by employing polymer micelles, immiscible solvent emulsions, inorganic or polymer (*e.g.*, polystyrene) particles, and bacterial or virus cells as templates; by etching solid silica particles; or by spray pyrolysis.^19–25^ Polymer micelles or emulsions provide very small particles, but making larger particles and tuning particle size are challenges in this approach. Similarly, the obtained particles typically fuse with one another, and achieving individually separated particles is a challenging task. Inorganic template etching is a time-consuming process, and in many cases, rudiments of inorganic templates remain in the hollow particle cavity if etching is incomplete. Unconventional techniques such as spray drying are inexpensive, but particle size control is difficult. The use of polystyrene particles as templates is attracting much attention because polystyrene particles can be synthesized at low cost with controlled sizes. Polystyrene particle-based synthesis of hollow silica particles involves three steps: (1) synthesis of polystyrene particles, (2) deposition of silica shells on polystyrene particles, and (3) removal of the polystyrene core by burning or dissolving to obtain hollow silica particles.

Synthesis of hollow silica particles having shells made of silica alone (pristine hollow particles) is well reported. Some previous efforts have been made to attach surfactant molecules to the surfaces of mesoporous (not hollow) hollow particles. For example, Zhang *et al.*^26^ first made porous silica particles by using cetyltrimethylammonium bromide (CTAB) as the template. In the next step, sodium carbonate-based etching was used to create cavities inside the porous particles, thus leading to porous-hollow silica particles. Then, 3-mercaptopropyl-trimethoxysilane (MPTS) was used to attach thiol-group ending surfactants to the surface. Similarly, Ribeiro *et al.*^27^ coated solid silica particles with poly(butyl methacrylate) to make superhydrophobic coatings. Similarly, hollow polymer particles have been reported by depositing a polymer layer around solid silica particles, followed by etching the silica core. The same hollow polymer particles were also converted to hollow carbon particles by pyrolysis of polymer.^28,31^ However, in this work, shell is made of a single material – polymer or carbon.^28,31^ To the best of our knowledge, no work has reported hollow particles with a hybrid shell – shell made of two layers of different materials (inner layer: silica and outer layer: polymer or carbon). Additionally, no previous report has investigated the effect of such an additional layer on the properties of the hollow silica particles. We envisage that such additional layers can change the properties, such as stability against moisture and thermal conductivity, of pristine hollow silica particles.

We report the synthesis of hybrid hollow silica particles, characterize these hybrid particles, and compare their properties with the properties of pristine hollow silica particles. Our investigations reveal that by changing the coating material, several intrinsic properties of hollow silica particles can be modified.

Hollow silica particles were synthesized by modifying previously reported strategies based on the use of polystyrene particles (synthesis details in ESI S1†) as a template.^1^ For synthesizing hollow silica particles, in a typical experiment, 0.25 g of polystyrene particles were mixed into 100 mL of ethanol/water (ethanol 80 mL, water 20 mL). A suitable amount of tetraethyl orthosilicate was added to make complete shells around the polystyrene particles. To increase the TEOS hydrolysis, 28–30% of ammonium hydroxide was used as a catalyst.

Fig. 1a depicts a schematic of hollow particle formation. Fig. 1b shows an SEM image of the polystyrene particles used as templates, and Fig. 1c shows a transmission electron microscope (TEM) image of polystyrene core-silica shell particles. Fig. 1d shows an SEM and Fig. 1e shows a high-angle annular dark-field scanning transmission electron microscopy (HAADF-STEM) image of hollow silica particles obtained after burning the polystyrene core by keeping the sample at 550 °C for 4 h.

**Fig. 1:**
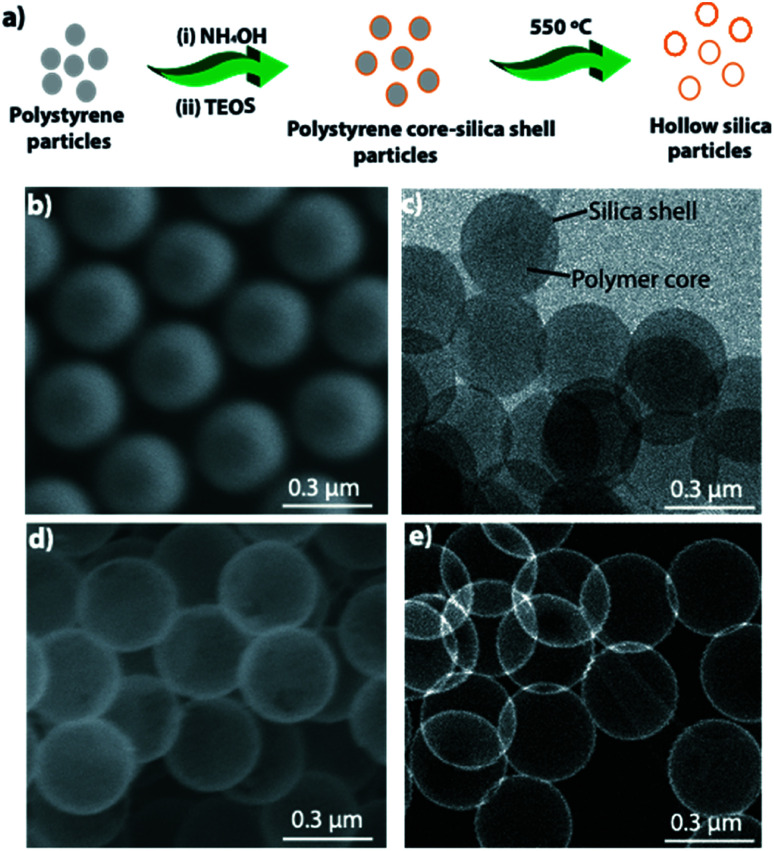
(a) Schematic showing synthesis of hollow silica particles. (b) SEM image of polystyrene particles. (c) TEM image of polystyrene core coated with silica shell (core–shell). (d) SEM and (e) HAADF-STEM image of hollow silica particles.

There are several polymers that can be used to form coatings on silica.^28–31^ Among these, the use of resorcinol is well studied.^28,31^ In a typical experiment, 0.25 g of hollow particles (0.25 g) were mixed in water (100 mL). Ammonium hydroxide (28–30%, 500 μL), resorcinol (0.1 g), and formaldehyde (150 μL) were added to this reaction mixture. The reaction was allowed to proceed overnight (≈16 h) to completion. Expected mechanism for polymer coating formation is explained in ESI S3.†Fig. 2a shows a schematic of the process used to make a polymer (polyresorcinol) coating on a silica shell. Fig. 2b shows low-magnification (i) and high-magnification (ii) TEM images of polymer-coated hollow silica particles. The polymer coating can be clearly seen (light in contrast) around the silica shell (dense in contrast). Though TEM imaging confirmed the presence of a polymer coating on the surface of the silica, to further confirm the formation of the coating, we applied electron energy loss spectroscopy (EELS). Energy dispersive X-ray (EDX) imaging is easy to use and is a readily available technique for analysing materials; however, EDX has a very low sensitivity to low-atomic-weight elements such as carbon and oxygen. Therefore, it was not a suitable technique for confirming the polymer presence. In contrast, EELS is known for its high sensitivity to low-atomic-weight elements (*e.g.*, carbon and oxygen). Fig. 2c shows scanning HAADF-STEM (i) and EELS (ii) images of the polymer-silica hybrid shell. The coating was quite uniform, with some thicker areas on the free surfaces of particles and some thinner areas at the joints in aggregated particles (ESI S2†). Therefore, if individual uniform coatings are required, the original hollow particle samples must be properly disaggregated.

**Fig. 2:**
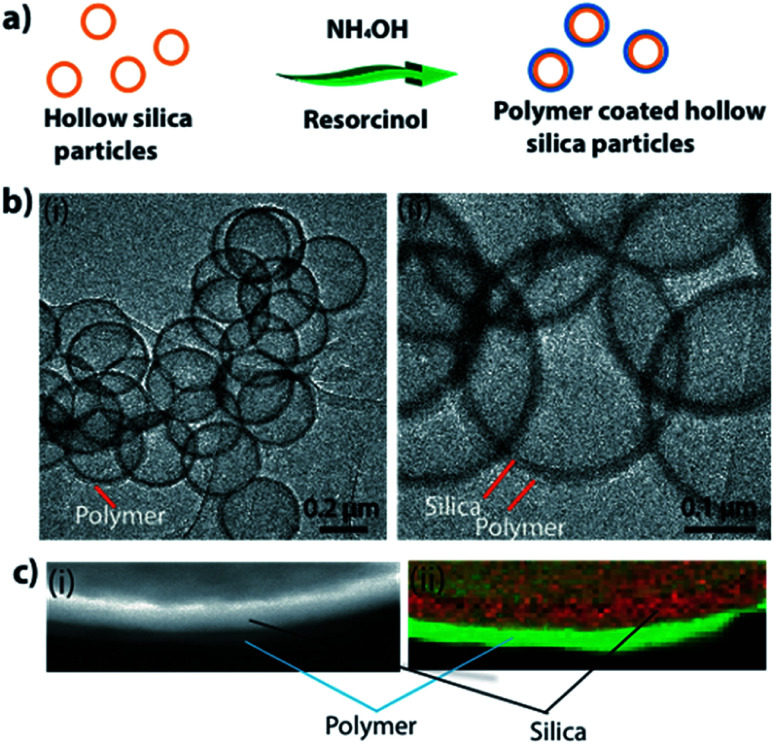
(a) Schematic showing the polymer coating process. (b) TEM images of polymer-coated silica particles. (c) HAADF-TEM (i) and EELS S map (ii) showing polymer and silica layers of hybrid shell.

Additionally, we demonstrated the formation of hybrid hollow silica particles with outer layers made of carbon and inner layers made of silica. To form a carbon layer on a silica shell, the initial polymer coating was sintered in an inert atmosphere (argon) at 550 °C for 4 h. Fig. 3a shows a schematic of polymer layer conversion to a carbon layer. Under these conditions, polymer converts into carbon instead of being completely oxidized into carbon dioxide and water. After heating under an inert atmosphere, brown polymer-coated particles changed to black carbon-coated particles. Separate carbon (outer) and silica (inner) layers were observed in TEM (Fig. 3b) and EELS images (Fig. 3c).

**Fig. 3:**
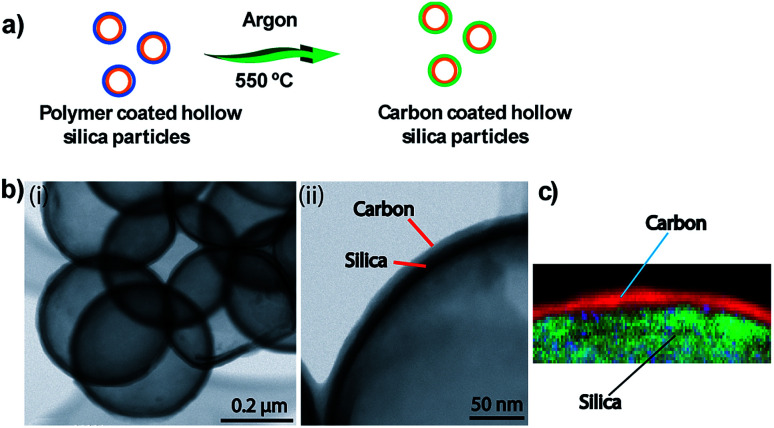
(a) Schematic showing conversion of polymer coating to carbon coating. (b) TEM images of carbon-coated particles. (c) EELS element map showing the carbon layer on a silica shell.

In addition to making hybrid shell hollow particles, we investigated whether the coating affected the properties (*e.g.*, thermal conductivity and moisture absorption) of the pristine hollow silica particles. We measured the thermal conductivity of pristine, polymer-coated, and carbon-coated particles. The results showed that polymer-coated particles had the lowest thermal conductivity and carbon-coated particles had the highest thermal conductivity of the three types. The Fig. 4 plot shows the thermal conductivities of the three types of particles, and respective insets show photos of corresponding particle samples. More details of the measurements are provided in ESI-S3.† As expected, the polymer silica particles had lower thermal conductivity (0.022 ± 0.002 W m^−1^ K^−1^) than pristine hollow particles (0.024 ± 0.002 W m^−1^ K^−1^), whereas carbon-coated particles had higher thermal conductivity (0.036 ± 0.004 W m^−1^ K^−1^) than both the pristine and the polymer-coated particles. This information provides a new tool to achieve or tune thermal properties of hollow silica particles as desired. For example, for high-thermal-insulation materials, polymer-coated particles are ideal; whereas carbon-coated particles are more suitable where somewhat higher thermal conductivity, but hydrophobicity is required. We were expecting that a carbon coating will increase electrical conductivity of hollow particles, however, we observed that even carbon coated particles had an electrical resistance in the megaOhm range, *i.e.*, behave as electrically insulators (measurement details in ESI S3†). Although the thermal conductivity of polymer-coated or carbon-coated hollow silica particles can be further modified by modifying the coating thickness, in the present work, we did not investigate the effect of coating thickness on thermal conductivity in detail. We expect the thinner the coating, the lower the thermal conductivity will be. We observed in both the polymer- and carbon-coated particles that the coatings were not uniform. Some particles had thick and others thin coatings, indicating that coating nucleation was not uniform, and the coatings may have begun forming earlier on some particles than on others. We observed that carbon–silica hollow particles are hydrophobic in nature, staying afloat on water for several hours (ESI Fig. S4†) and mixing in water only after vigorous stirring. It appears that, with stirring, water molecules enter the hollow particle cavities through the pores present in the carbon and silica shells and wet the inner parts of the cavities, thus causing the particles to mix in water.

**Fig. 4:**
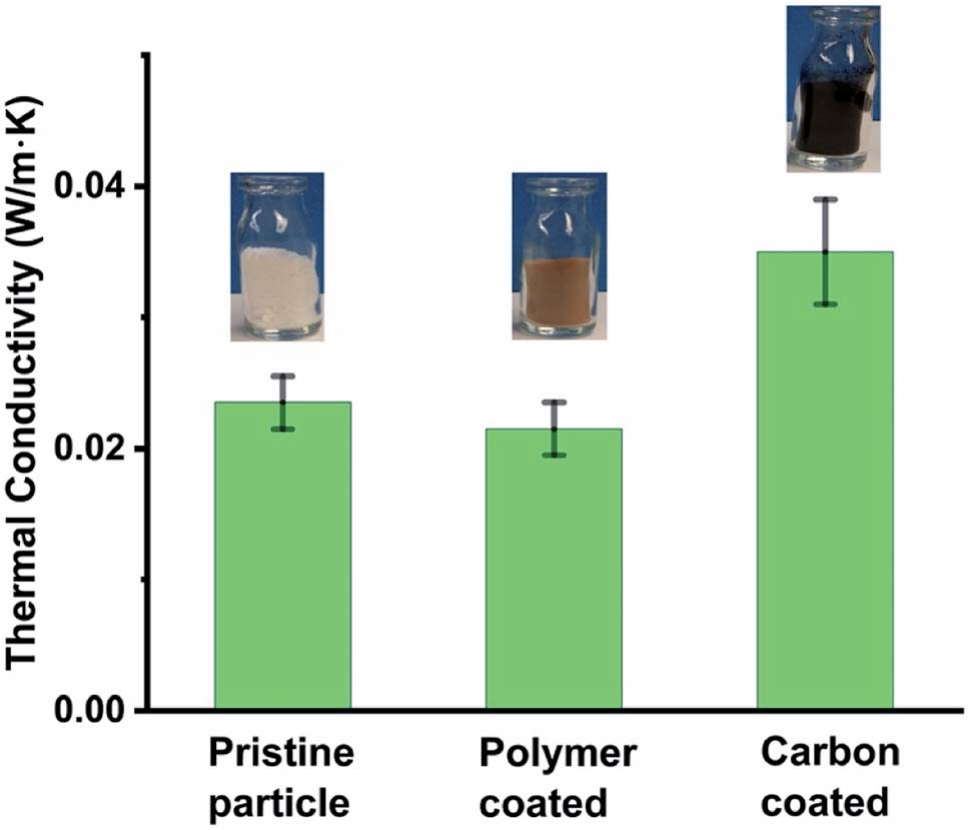
Effect of different types of coatings on the thermal conductivity of hollow silica particles. Insets show the photos of respective particles.

Additionally, we compared the moisture absorption properties of pristine hollow silica particles with those of polymer- and carbon-coated hollow silica particles (Fig. 5). Moisture absorption/desorption experiments were performed using a dual vapor gravimetric sorption analyser. We observed that polymer-coated particles absorbed less humidity compared with pristine particles at the same relative humidity. However, both materials had similar isotherm profiles in which the moisture adsorption capacity increased at relatively higher moisture concentrations. The carbon-coated particles, on the other hand, showed a completely different isotherm behaviour: an immediate increase in adsorption capacity was observed between 30% and 50% relative humidity. A sharp increase in moisture absorption at higher relative humidity (between 30–50%) appears due to the entry of water vapors inside the particles because of porous nature of carbon layer. Similar shape of isotherms for pristine and polymer coated particles indicates that both of these particles had similar surface groups (–OH), but lower absorption in polymer coated particles compared to pristine particles indicates that its surface has a small number of moisture absorbing groups (–OH) compared to pristine particles. The hysteresis between adsorption and desorption isotherms was found to be minimal, indicating that the samples had similar performance for adsorption or desorption process. We expect this information to be helpful for applications such as developing water-stable coatings or insulation materials by using hollow silica particles.

**Fig. 5:**
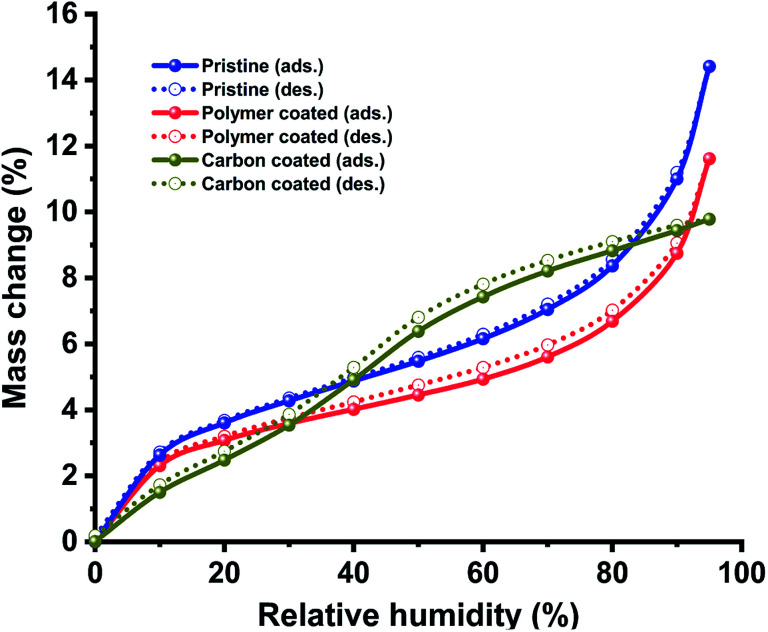
Effect on moisture adsorption and desorption process. Plot showing behaviour of hollow particles under different relative humidity conditions for pristine and coated samples.

## Conclusions

We demonstrated a strategy to synthesize two types of hybrid hollow silica particles having shells consisting of either (1) an outer layer of polymer and an inner layer of silica, or (2) an outer layer of carbon and an inner layer of silica. Additionally, we compared the properties of pristine, polymer-, and carbon-coated hollow silica particles. Polymer-silica particles had low thermal conductivity and absorbed less moisture than the pristine hollow silica particles. Particles having shells of carbon–silica had higher thermal conductivity and were somewhat hydrophobic compared with the pristine hollow silica particles. These studies provide insights into how additional different material layers modify the properties of hollow silica particles and can be further applied to other types of materials such as rods, solid particles, or fibres. We anticipate that these hybrid particles will open new opportunities for fabricating thermal insulation materials, superhydrophobic coatings, and drug delivery carriers.

## Conflicts of interest

There are no conflicts to declare.

## Supplementary Material

RA-010-D0RA02888F-s001
