# Supplementary material for: Hybrid hollow silica particles: synthesis and comparison of properties with pristine particles
Source: RSC Adv. 2020 Jun 10;10(38):22331–4. doi: 10.1039/d0ra02888f (PMC9054576; doi:10.1039/d0ra02888f)
Supplement: RA-010-D0RA02888F-s001 [file RA-010-D0RA02888F-s001.pdf]

Electronic Supplementary Material (ESI) for RSC Advances.
This journal is © The Royal Society of Chemistry 2020

Electronic Supplementary Information

Hybrid hollow silica particles: Synthesis and comparison of properties with pristine particles

Jaswinder Sharma* David A. Cullen, Georgios Polizos, Kashif Nawaz, Hsin Wang, Nitin Muralidharan, and David Barton Smith

S1:

Chemicals:

Styrene, tetraethyl orthosilicate (TEOS), ammonium hydroxide (28-30%), resorcinol, and formaldehyde solution were purchased from Sigma Aldrich. 2,2'-Azobis(2-methylpropionamidine) dihydrochloride was purchased from ACROS-organics through fisher scientific.

Synthesis of polystyrene particles:

In a typical experiment, 2.0 mL of styrene was added to 100 mL of water. The reaction mixture was heated for 20 min at 65°C. Then 160 mg (dissolved in water) of 2,2'-Azobis(2-methylpropionamidine) dihydrochloride was added to the stirring reaction mixture, and the reaction was allowed to continue for 10 h. Particles were characterized by scanning electron microscopy (SEM), and very nice monodisperse particles approximately 300 nm in diameter were obtained. Figure S1. Shows the SEM images of polystyrene particles.

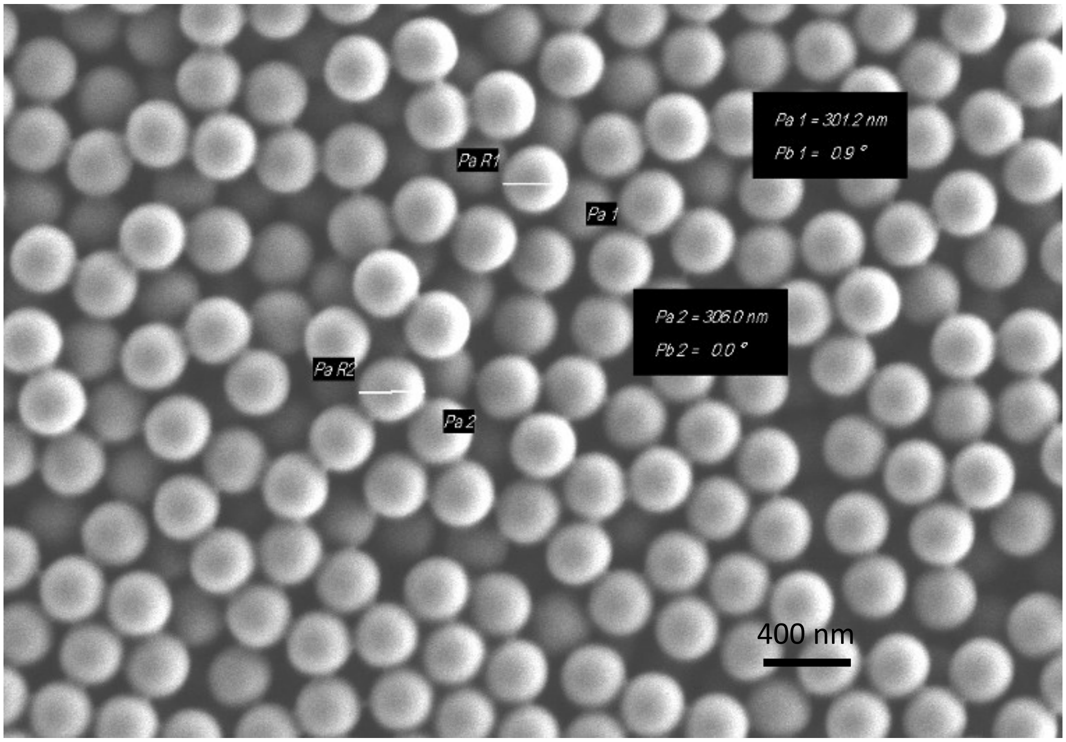

Figure S1: SEM images of polystyrene particles.

Synthesis of hollow silica particles:

In a typical experiment, 0.25 g of polystyrene particles were mixed into 100 mL of ethanol/water (ethanol 80 mL, water 20 mL). 200 µL of tetraethyl orthosilicate was added to make shells around the polystyrene particles. To increase the TEOS hydrolysis, 28–30% of ammonium hydroxide was used as a catalyst. The obtained core-shell particles were heated at 550 ºC for 4 h in order to remove the polystyrene core and obtain the hollow silica particles. For obtaining large amounts of hollow particles for thermal conductivity measurements, synthesis size was proportionately increased. Figure S2 shows the SEM images of hollow silica particles.

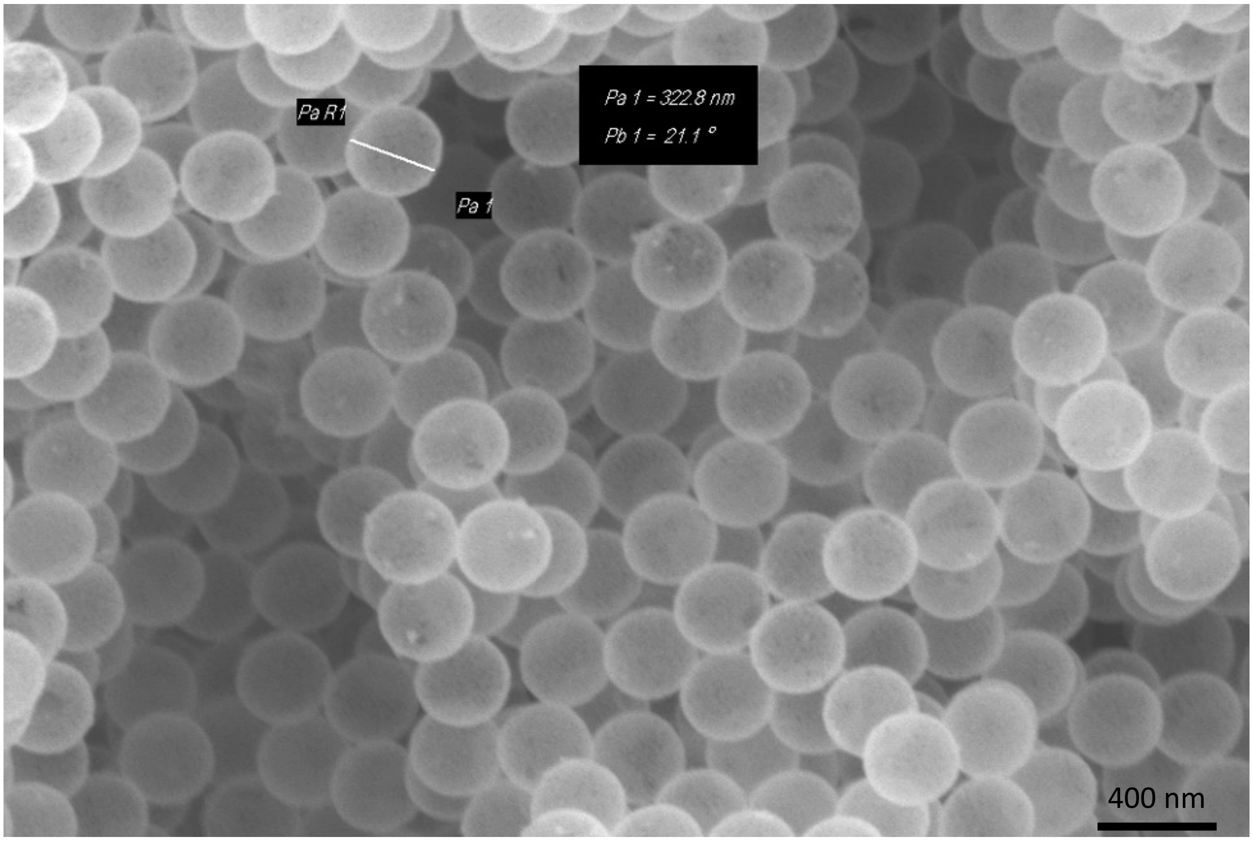

Figure S2: SEM images of hollow silica particles.

S2:

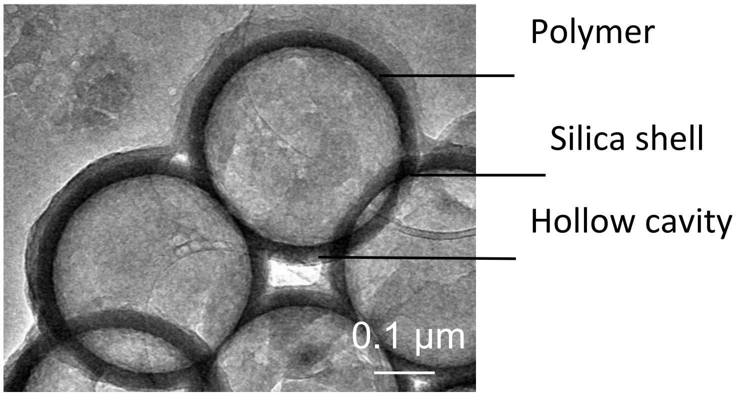

Figure S3. Showing the thin polymer coating at joints and thick polymer coating at free particle surface.

Thermal conductivity measurements:

Thermal conductivity measurements were performed by using TPS 2500 S instrument by using a sensor C5465. Sample holder provided by manufacturer was used, and no weight was added on the samples, i.e., thermal conductivity measurements were performed on loose particles without any pressing.

Additionally, we observed in all samples, the thermal conductivity is lowest when sample is dry. Keeping the samples in moisture enhanced their thermal conductivity considerably. Therefore, all thermal conductivity measurements were made by drying the samples 80 ºC for overnight. Figure S4 shows the setup used for measuring the thermal conductivity.

Possible effect of packing density:

Additionally, packing density of particles can also impact the final thermal conductivity of hollow particles, i.e., higher the packing density, the higher the thermal conductivity. To avoid this discrepancy, we tried our best to keep the experimental conditions similar for all the three samples (hollow, polymer, and carbon coated). Means, drying, putting no weight on samples, making the fine powder by lightly mashing the samples in crucibles etc. Additionally, we measured thermal conductivities on several samples prepared from different original hollow particles, and the thermal conductivity values trend remained same (polymer coated < pristine hollow < carbon coated).

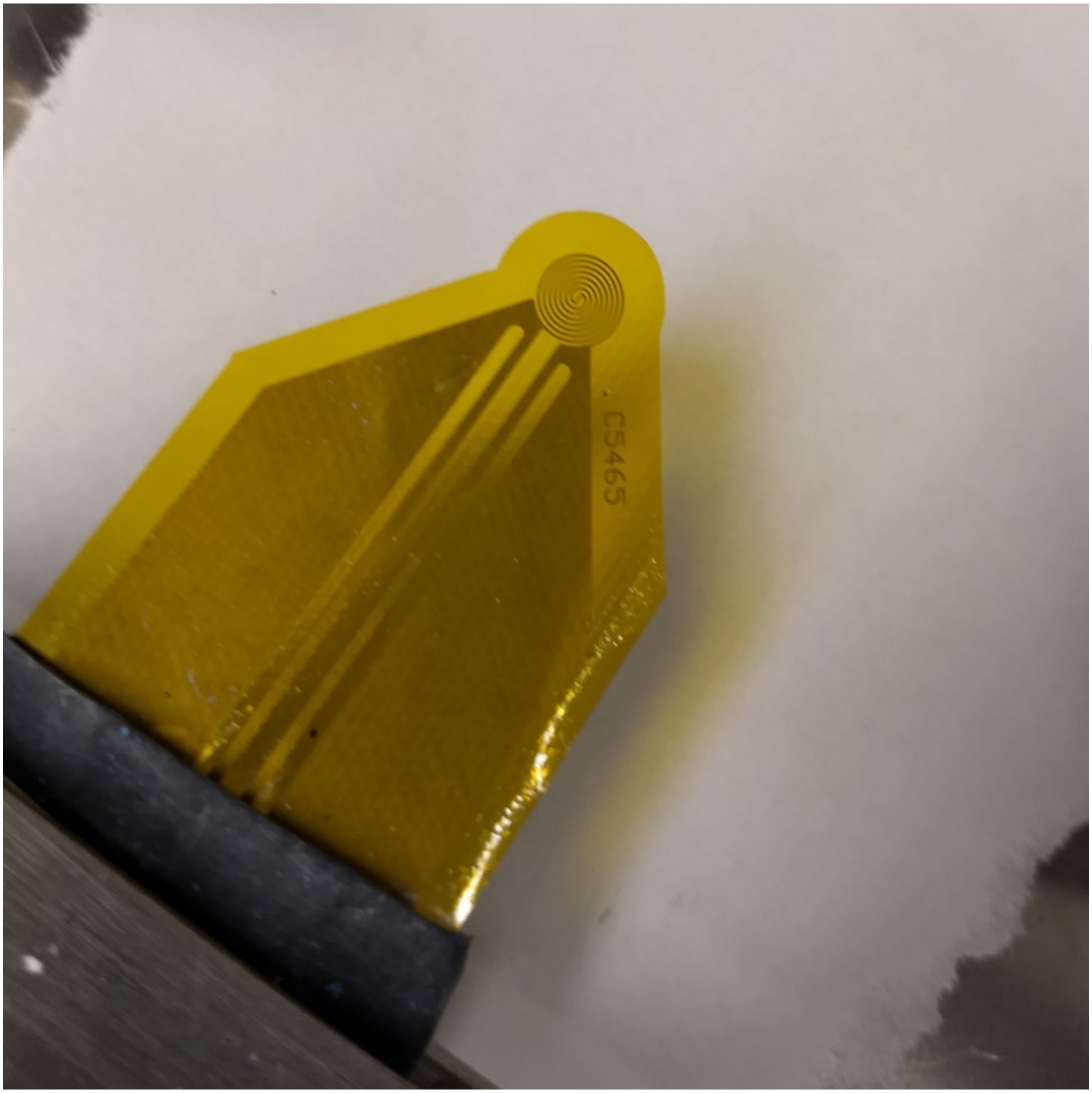

Sensor used for the thermal conductivity measurements.

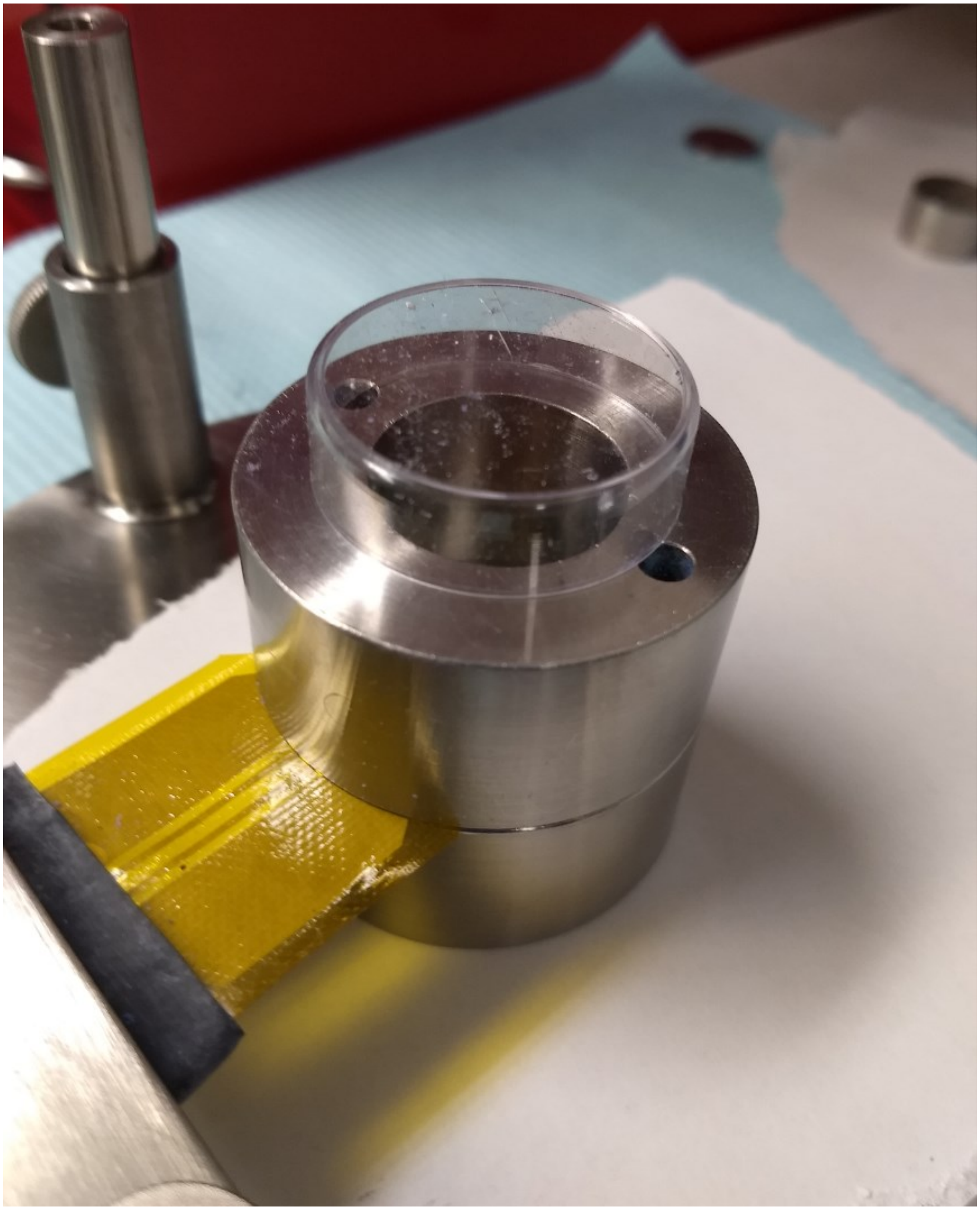

sensor and the setup used for measuring the thermal conductivity.

S3.

Expected mechanism of polymer shell formation.

From previous reports[1] on polymer coating formation on solid silica particles, we assume that initially NH_4^+ ions stick to the hollow silica particle surface and render it somewhat +ve charge. In the meantime, in the solution resorcinol and formaldehyde reaction occurs, catalyzed by OH^- ions, which results in small oligomers of resorcinol-formaldehyde polymerized structures having -OH groups. Due to electrostatic attractions between -OH groups on these structures and NH_4^+ ions on particle surface, these oligomers diffuse to the hollow silica particle surfaces. Later on condensation between these oligomers results in the formation of polymer shell around hollow silica particles. *Ref. 1. Provides more details.*

Electrical conductivity measurement:

First a pallet of carbon coated particles was made by manually pressing. The electrical resistance of the pallet was measured using a Keithley 2410-C source meter. Measurements were carried out using a two-probe configuration. The resistance of the sample was higher than one Megaohm (MW). Indicating carbon coating didn't help much in lowering the resistance (or in increasing the electrical conductivity) of the hollow particles.

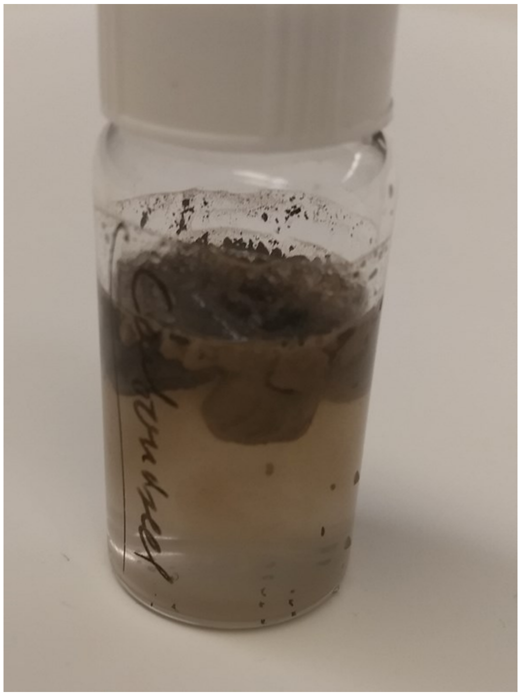

Figure S4. shows photo of carbon coated particles floating in water.

S4.

Estimates of polyresorcinol/hollow particle

Average thickness of polyresorcinol layer ≈ 7 nm
Hollow particle radius ≈ 322/2 ≈ 161 nm
Hollow particle with polymer coating radius ≈ 329/2 ≈ 164.5 nm

Volume of polyresorcinol/particle ≈ $4/3\ \pi\ [R^3 - r^{3]} = 4/3\ \times 3.14\ [(164.5)^3 - (161)^3]\ nm^3$

$\approx 4/3\ \pi\ [R^3 - r^{3]} = 4/3\ \pi\ [(322)^3 - (315)^3]\ nm^3$
$\approx 1.16 \times 10^{-21}\ nm^3$
$\approx 1.16 \times 10^{-15}$ c
Density of polysresorcinol ≈ $1.28\ mg/cm^3$
Mass of polyresorcinol/particle ≈ $1.16 \times 10^{-15}\ cm^3 \times 1.28\ mg/cm^3$ = **1.48×10^{-15} mg/ particle**

Estimate of carbon/hollow particle

To estimate, the amount of carbon/particle, we first made polymer coating around hollow particles, and then measured the percentage of weight loss during pyrolysis.

Hollow silica particles: 250 mg
Polymer coated particles: 313 mg ≥≥ Polymer weight: 63 mg
After pyrolysis the weight: 283 g ≥≥ or carbon weight 33 mg

% weight of carbon after carbonization process: 33/63 x 100 ≥≥ 52.38

Therefore, estimated weight of carbon/particle ≈ 1.48×10^{-15} mg x 0.5238 >> **7.75×10^{-16} mg**

Ref.

1. A. B. Fuertes et. al. *Chem. Commun.,* **2012,** *48*, 6124-6126.
